# *Gan-jiang-ling-zhu* decoction improves steatohepatitis by regulating gut microbiota-mediated 12-tridecenoic acid inhibition

**DOI:** 10.3389/fphar.2024.1444561

**Published:** 2024-08-23

**Authors:** Ruohui Xu, Jiaxuan Wu, Jiashu Pan, Shengan Zhang, Yunuo Yang, Li Zhang, Wenjun Zhou, Na Wu, Dan Hu, Guang Ji, Yanqi Dang

**Affiliations:** ^1^ Institute of Digestive Diseases, China-Canada Center of Research for Digestive Diseases (ccCRDD), Shanghai University of Traditional Chinese Medicine, Shanghai, China; ^2^ Department of Traditional Chinese Medicine, School of Medicine, First Affiliated Hospital, Zhejiang University, Hangzhou, Zhejiang, China; ^3^ State Key Laboratory of Integration and Innovation of Classic Formula and Modern Chinese Medicine (Shanghai University of Traditional Chinese Medicine), Shanghai, China; ^4^ School of Public Health, Shanghai Innovation Center of Traditional Chinese Medicine Health Service, Shanghai University of Traditional Chinese Medicine, Shanghai, China; ^5^ Seventh People’s Hospital of Shanghai University of Traditional Chinese Medicine, Shanghai, China

**Keywords:** nonalcoholic steatohepatitis, gan-jiang-ling-zhu decoction, gut microbiota, 12-tridecenoic acid, acetyl-coenzyme A carboxylase alpha

## Abstract

**Introduction:** *Gan–jiang–ling–zhu* (GJLZ) decoction is a classical traditional Chinese medicine prescription. Through invigorating *yang*, activating *qi* and dissipating *dampness*, GJLZ decoction is widely applied for the treatment of chronic digestive disease, including nonalcoholic fatty liver disease. However, efficacy and mechanism of GJLZ decoction behind nonalcoholic steatohepatitis (NASH) treatment remains unelucidated.

**Methods:** NASH was induced in mice, followed by treatment with GJLZ decoction. Various methods including hematoxylin-eosin, oil red O staining, and triglyceride analysis were employed to evaluate the treatment effects of GJLZ decoction on NASH. Gut microbiota, metabolomics, cell viability assays, immunofluorescence and Western blotting were performed to unveil the mechanism behind GJLZ decoction.

**Results:** GJLZ decoction treatment significantly improved hepatic steatosis in mice with NASH. It led to remodeling of gut flora and metabolite structures, including the 12-tridecenoic acid level. 12-Tridecenoic acid aggravated hepatic steatosis by promoting acetyl-coenzyme A carboxylase alpha (ACC) expression and inhibiting carnitine palmitoyltransferase 1A (CPT1A) expression. GJLZ decoction treatment reduced the 12-tridecenoic acid level, inhibited ACC activity and promoted CPT1A expression.

**Conclusion:** Our results demonstrated that 12-tridecenoic acid aggravated hepatic steatosis by affecting the ACC–CPT1A axis and GJLZ decoction treatment effectively reduced the 12-tridecenoic acid level and improved steatosis.

## 1 Introduction

The prevalence of nonalcoholic fatty liver disease (NAFLD), a systemic metabolic disease, is increasing and it significantly impacts approximately one third of the whole world population (Younossi et al., 2019; Lazarus et al., 2022). NAFLD is a worldwide prominent contributor to chronic liver diseases, with many patients progressing to nonalcoholic steatohepatitis (NASH). As NASH advances, inflammation and progressive liver damage can result in cirrhosis and even hepatocellular carcinoma. This ultimately makes NAFLD the primary reason behind end-stage liver disease (Younossi, 2019). NAFLD thus places a substantial burden on public health (Friedman et al., 2018).

The pathogenesis and progression of NAFLD are not yet fully understood. “Two hit” theory cannot explain the multiple physiological and pathophysiological effects of NAFLD (Buzzetti et al., 2016). Variation in primary disease drivers and modifiers causes patients with NAFLD to have heterogeneous disease severity and medical history (Eslam et al., 2020). These factors make it difficult to discover an active drug for treatment. Some traditional Chinese medicine prescriptions are effective for treating NAFLD. According to our previous research, the administration of *ling–gui–zhu–gan* decoction can improve liver steatosis and NASH through regulating N6-methyladenosine or gut flora and metabolites (Dang et al., 2019; Dang et al., 2020a; Zhu et al., 2023). In addition, *ling–gui–zhu–gan* decoction treatment improved the insulin sensitivity of patients with obesity with NAFLD in a clinical study (Dai et al., 2022). *Jiangzhi* granule treatment can improve NASH by regulating the levels of bile acid and gut flora (Li, C. et al., 2021; Cao et al., 2022). By modulating the abundance of gut flora and serum metabolites, *Dachaihu* decoction treatment ameliorates NAFLD (Cui et al., 2020). In these studies, the efficacy of traditional Chinese medicine (TCM), gut flora, and metabolites have been demonstrated in the treatment of NAFLD.

*Gan–jiang–ling–zhu* (GJLZ) decoction is a classic TCM formula originated from *Synopsis of Golden Chamber* (Jin Kui Yao Lüe) about two thousand years ago and is composed of *Glycyrrhiza uralensis* Fisch. (Gancao), *Zingiber officinale* Rosc. (Ganjiang), *Poria cocos* (Schw.) Wolf (Fuling), and *Atractylodes macrocephala* Koidz. (Baizhu), and mixed at a ratio of 1:2:2:1. GJLZ decoction is widely applied clinically for invigorating *yang*, activating *qi*, dissipating dampness and strengthening spleen (Liu et al., 2020), and chronic digestive diseases (Wang, 2016; Li, X. et al., 2021; Dai and Ji, 2023). GJLZ decoction and its effective components could alleviate hepatic steatosis, liver injury and inflammation, and dysfunction of metabolism (Ezzat et al., 2018; Oliveira et al., 2019; Dang et al., 2020b; Gou et al., 2021; Shi et al., 2022; Ye et al., 2022; Chen et al., 2023; Zhang et al., 2023; Tang et al., 2024). However, the efficacy of GJLZ decoction treatment on NASH remains unknown. The purpose of this study is to investigate the effects of GJLZ decoction on NASH treatment in mice that are fed a methionine- and choline-deficient (MCD) diet. Profiles of gut flora and metabolite were performed and analyzed. The results revealed that GJLZ decoction treatment remodeled the gut flora and metabolite structures, and reduced the level of 12-tridecenoic acid. Additionally, 12-tridecenoic acid aggravated hepatic steatosis by promoting acetyl-coenzyme A carboxylase alpha (ACC) activity, and GJLZ decoction treatment inhibited ACC activity. The findings demonstrated that treatment with GJLZ decoction improved NASH by affecting the gut flora–mediated 12-tridecenoic acid inhibition.

## 2 Materials and methods

### 2.1 GJLZ decoction preparation

The GJLZ decoction comprises extracts of *Glycyrrhiza uralensis* Fisch. (Gancao), *Zingiber officinale* Rosc. (Ginger), *Poria cocos* (Schw.) Wolf (Fuling), and *Atractylodes macrocephala* Koidz. (Baizhu), and mixed at a ratio of 1:2:2:1, respectively. Formula granules of these four ingredients were supplied by Longhua Hospital. The fingerprint region of the infrared spectrum of the GJLZ decoction was determined using ultra-performance liquid chromatography followed by quadrupole time-of-flight mass spectrometry. Briefly, GJLZ decoction was dissolved using 40% methanol solution, and extracted by ultraphonic (100 W, 40 kHz) for 30 min. A centrifuge was then used to spin the sample for 15 min at 12,000 rpm. A supernatant was then collected from the centrifuge. The mobile phase was pumped at a flow rate of 0.3 mL/min and the analytes were separated.

### 2.2 Animal model

**FIGURE 1:**
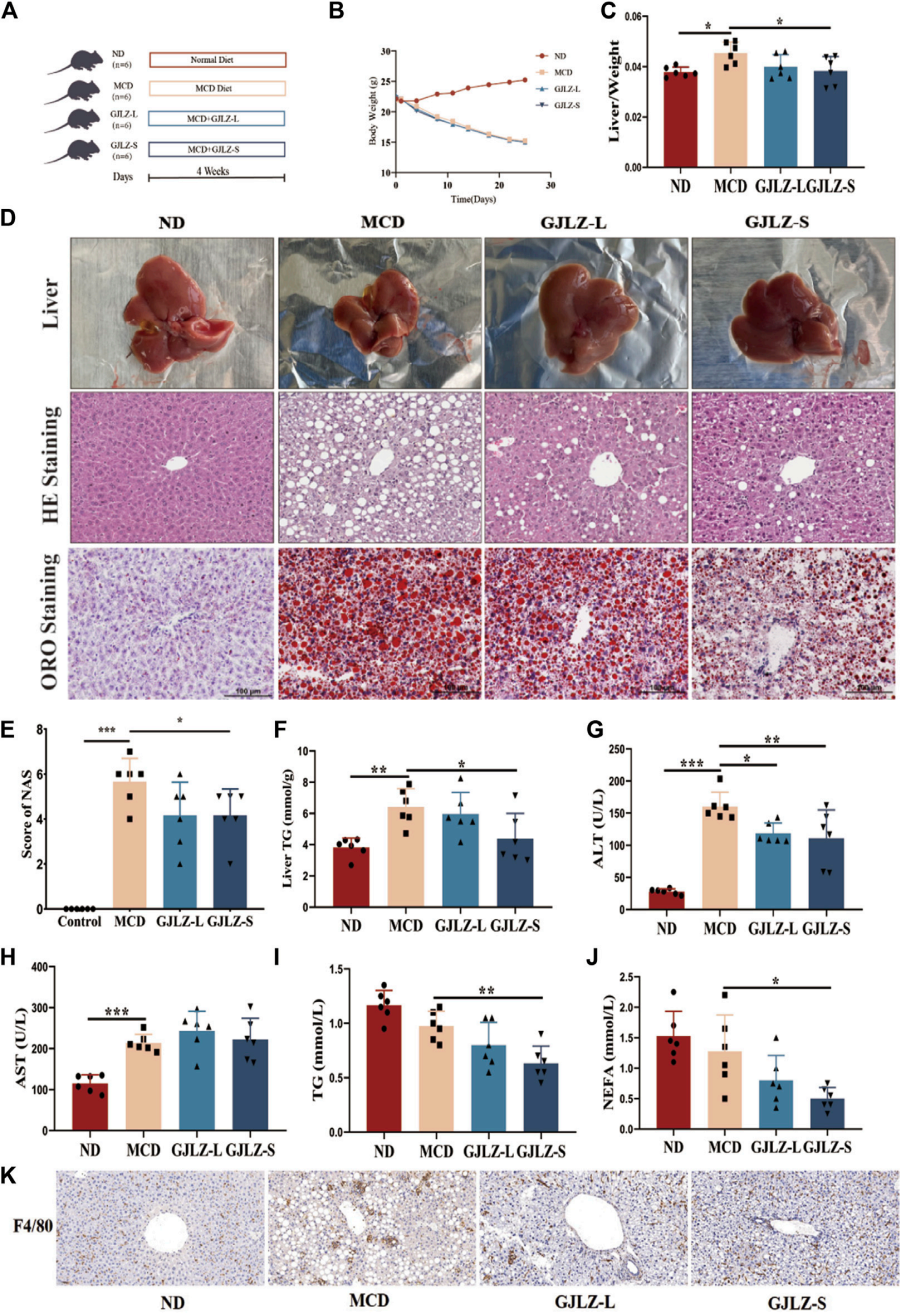
GJLZ decoction treatment improved hepatic steatosis in mice induced by methionine- and choline-deficient (MCD) diet. **(A)** Study flowchart. **(B)** Mice weight and **(C)** Liver/body weight ratio was shown after GJLZ decoction treatment. **(D)** Hematoxylin-eosin, oil red O staining and **(E)** Nonalcoholic fatty liver disease activity scores were performed after GJLZ decoction treatment. **(F)** Level of TG were performed after GJLZ decoction treatment. **(G–J)** Serum levels of alanine transaminase, aspartate transaminase, TG, and nonesterified fatty acid were measured. **(K)** IHC for F4/80 reflects the degree of inflammation in the liver. Data presented as the mean ± standard deviation (SD) (n = 6). ^*^ *p* < 0.05, ^**^ *p* < 0.01, ^***^ *p* < 0.001.

Male C57BL/6J mice, aged 6 weeks, were obtained from Shanghai Laboratory Animal Center (SLAC) and maintained standard facility. Twenty-four mice were randomly divided into four groups: normal diet group (ND), MCD group, low-dose GJLZ decoction (GJLZ-L) and standard-dose GJLZ decoction (GJLZ-S). The ND group received a normal diet for 4 weeks, while the MCD group was treated with an MCD diet. The GJLZ-L group received an MCD diet along with GJLZ decoction (1.07 g/kg/d), and the GJLZ-S group received an MCD diet along with GJLZ decoction (2.14 g/kg/d) through gavage (Figure 1A). After 4 weeks, liver tissues were obtained, frozen or stored in formalin for pathological staining. Blood was acquired and serum was then separated. Serum and liver levels of total triglyceride (TG) and total cholesterol (TC) were measured.

### 2.3 Hematoxylin-eosin staining

Samples of liver tissue of the mice were immersed in 10% formalin for 48 h, underwent dehydration with ethanol, and subsequently embedded in paraffin wax. 4-μm slices of samples embedded by paraffin wax were sectioned. Samples were stained with hematoxylin-eosin kits. Photographs were captured by a microscope. Then NAFLD activity score (NAS) system was utilized to assess the histological scores of the liver.

### 2.4 Oil red O staining

Livers were embedded in OCT compound and frozen at −80°C. The frozen livers were sectioned at a thickness of 8 μm using a cryostat microtome maintained at −20°C. Permeabilization of the tissues was achieved using a 4% solution of paraformaldehyde. The samples were stained with Oil Red O buffer and hematoxylin and then the lipid droplets were observed with an inverted microscope.

### 2.5 Fecal 16S ribosomal DNA sequencing

Shanghai Applied Protein Technology (Shanghai, China) conducted sequencing of 16S ribosomal DNA from fecal samples. Briefly, magnetic soil and stool DNA kit were applied to extract genome DNA from feces (Tiangen, China), and then concentration and purity were verified. Subsequently, samples were amplified. Finally, sequencing libraries were obtained, and samples were subsequently sequenced. Analysis of data was performed using UPARSE software. Alpha and beta diversity was analyzed, including ACE, Chao1, Shannon and Simpson. Beta diversity was visualized using principal coordinate analysis (PCoA).

### 2.6 Targeted metabolomics

To extract the metabolites, 20 mg of fecal sample and 10 zirconium oxide beads were added to a mixture of 800 μL of methanol, acetonitrile and purified water. Homogenized for 3 min and then centrifuged for 20 min at 8,000 g rpm and the supernatant was taken and added to a 96-well plate. Add 20 μL of freshly prepared derivatization reagent to each well. The plates were sealed and derivatised at 30°C for 60 min and the samples were evaporated for 2 h. Finally, the standards and sample reconstituted in 50% ethanol were added to the 96-well plate for LC-MS analysis.

### 2.7 Cell culture and treatment

Alpha mouse liver 12 cells (AML12) and human hepatocellular carcinoma HepG2 were obtained from Shanghai Cell Bank. Primary hepatocytes were extracted from the liver of mice according to previous studies (Hu et al., 2018; Charni-Natan and Goldstein, 2020). Free fatty acids (FFAs) with or without 50 and 100 μM 12-tridecenoic acid were used to treat AML12 and HepG2 cells at a ratio of 2:1 (oleic acid and palmitic acid). Following a 24-h period, the cells were gathered, and both RNA and protein were extracted from them. In addition, ACC inhibition (TOFA, T3988, TargetMol, Shanghai, China) was used to explore the effects of 12-tridecenoic acid on steatosis.

### 2.8 Cell counting kit-8 assay

AML12 and HepG2 cells were seeded, and then various concentrations of 12-tridecenoic acid (12.5, 25, 50, 100, 200, or 400 μM) were applied to treat cells for 24 h. After removing the previous cell culture medium, the cell counting kit-8 was added. The cells were incubated at 37°C for 1 h, and measured at 450 nm.

### 2.9 Immunofluorescence

ZO-1 antibody (1:50, A0659, Abclonal, China) and occludin antibody (1:100, 91131, Cell Signaling Technology, United States) were used to detect intestinal permeability. After dewaxing, rehydrating, antigen rehydrating and blocking of endogenous peroxidase, samples of intestinal tract were incubated with ZO-1 and occludin antibodies at 4°C for 12 h. Subsequently, the samples were incubated in secondary antibodies at room temperature for 1 h. Finally, images of the specimens were captured.

### 2.10 Western blotting

The protein was obtained by utilizing RIPA lysis buffer and its concentration was measured. After separation, the protein was transferred, and the polyvinylidene fluoride membrane was blocked at room temperature for 1 h. Primary fatty acid synthase (1:1,000, FASN; ab22759, Abcam, United States), phospho-ACC (1:1,000, 3661, CST, USA), glucokinase (1:1,000, GCK; sc-17819, Santa Cruz Biotechnology, United States), ACC (1:1,000, 3676, CST, USA), carnitine palmitoyltransferase 1A (1:500, CPT1A; GTX114337, GeneTex, United States), carnitine palmitoyltransferase 1B (1:500, CPT1B; GTX117231, GeneTex, United States) antibodies, and β-actin antibody (1:10,000, ET1702-67, HuaAn Biotechnology) were obtained. Images were captured and subsequently analyzed using ImageJ to calculate optical density.

### 2.11 Statistical analysis

STAMP and LefSe software were utilized to analyze the abundances of individual metabolites and the quantitative analysis of biomarkers among the groups, respectively. The one-way ANOVA followed by Dunnet’s test was applied for significance determination. A *P*-value < 0.05 indicated significance.

## 3 Results

### 3.1 GJLZ decoction quality control

**FIGURE 2:**
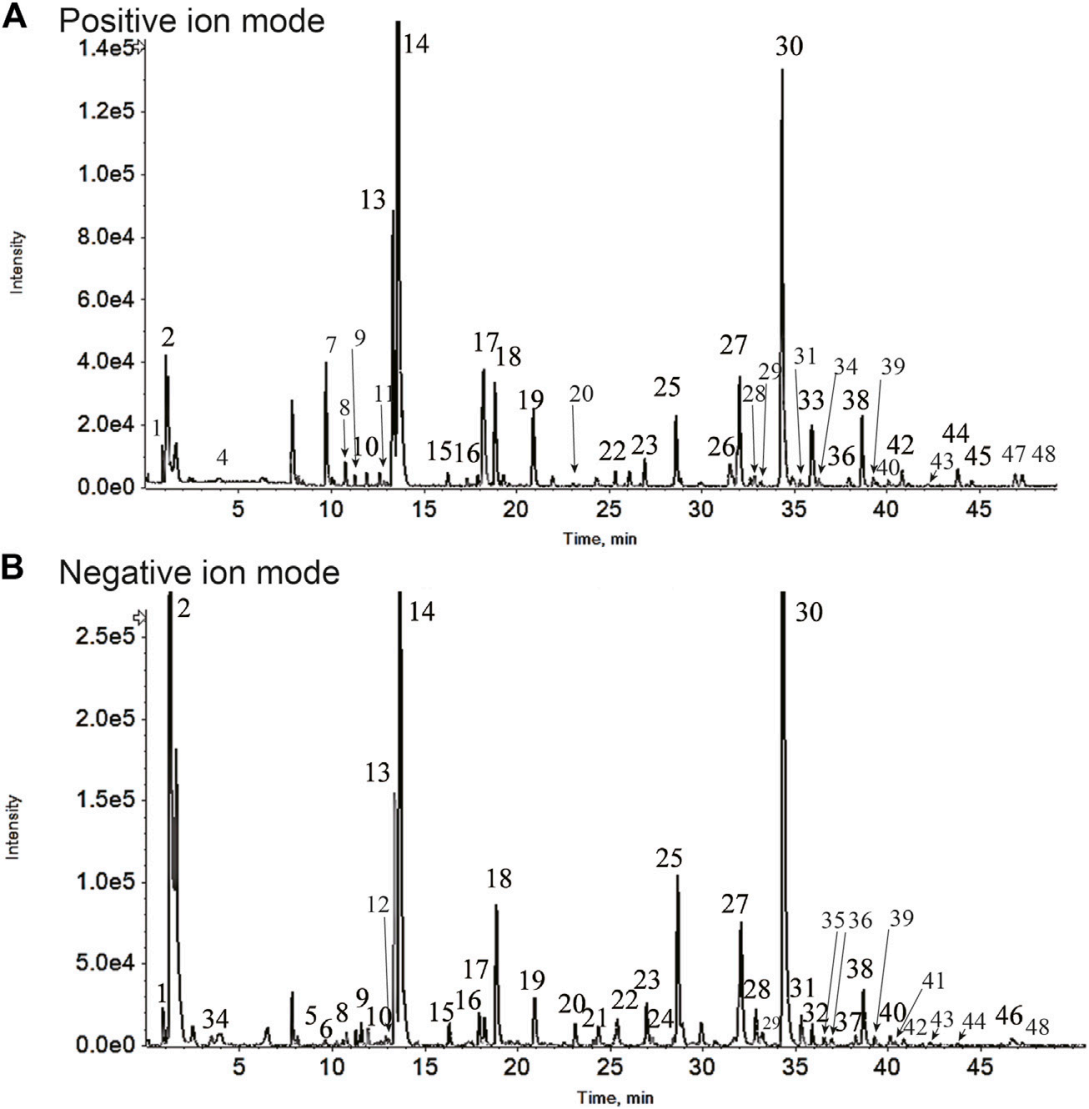
*Gan–jiang–ling–zhu* (GJLZ) decoction quality control was performed using ultra-performance liquid chromatography with quadrupole time of flight mass spectrometry. The results of the positive **(A)** and negative **(B)** ion mode analyses are show respectively. 1) Gluconic acid; 2) Sucrose; 3) Citric acid; 4) N-Fructosyl pyroglutamate; 5) Phenylalanine; 6) Atractyloside A; 7) L-Tryptophan; 8) 5-Hydroxyferulic acid; 9) Nicotiflorin; 10) Schaftoside; 11) Violanthin; 12) Licuraside; 13) Liquiritin apioside; 14) Liquiritin; 15) Naringenin 7-glucoside; 16) Isoliquiritin apioside; 17) Ononin; 18) Isoliquiritin; 19) Liquiritigenin; 20) Licorice saponin J2; 21) Hexahydrocurcumin; 22) Uralsaponin F; 23) Licorice saponin A3; 24) 22-Acetoxyl-rhaoglycyrrhizin; 25) 22β-Acetoxyl glycyrrhizic acid; 26) Licorice saponin E2; 27) Licorice saponin G2; 28) 22-β-Acetoxyglycyrrhetaldehyde; 29) Rhaoglycyrrhizin; 30) Glycyrrhizin; 31) Licoricesaponin B2; 32) Uralsaponin B; 33) 6-Gingerol; 34) Licoricesaponin K2; 35) Apioglycyrrhizin; 36) Atractylenolide Ⅰ; 37) Licoricesaponin C2; 38) Glycycoumarin; 39) Glyasperin C; 40) Licoisoflavone A; 41) Glycyrrhetinic acid 3-O-mono-beta-D-glucuronide; 42) Glycyrin; 43) Glycyrol; 44) AtractylenolideⅡ; 45) 6-Shogaol; 46) Poricoic acid B; 47) Poricoic acid A; 48) 16α-Hydroxydehydrotrametenolic acid.

The active components of GJLZ decoction were investigated. In total, 48 ingredients were verified (Figure 2; Table 1), mainly including sucrose, liquiritin apioside, liquiritin, ononin, isoliquiritin, liquiritigenin, 22β-acetoxyl glycyrrhizic acid, licorice saponin G2, glycyrrhizin, 6-Gingerol, atractylenolide Ⅰ, atractylenolide II, et al.

**TABLE 1:** Active ingredients of GJLZ decoction.

No.	Time (min)	Polarity	*m/z*	*ppm*	Formula	MW	Name	MS/MS
1	1.106	[M-H]^−^	195.0502	−4.10	C_6_H_12_O_7_	196.16	Gluconic acid	96.9599; 78.9585
2	1.23	[M-H]^−^	341.1078	−3.22	C_12_H_22_O_11_	342.3	Sucrose	119.0343; 89.0234; 59.0133
3	3.466	[M-H]^−^	191.0193	−2.09	C_6_H_8_O_7_	192.12	Citric acid	111.0081; 87.0084; 85.0292
4	3.96	[M-H]^−^	290.0875	−2.07	C_11_H_17_NO_8_	291.1	N-Fructosyl pyroglutamate	200.0569; 128.0348
5	8.338	[M-H]^−^	164.0717	0.00	C_9_H_11_NO_2_	165.08	Phenylalanine	147.0453; 103.0556; 72.0092
6	9.62	[M+HCOO]^−^	493.2279	−2.43	C_21_H_36_O_10_	448.23	Atractyloside A	447.2220; 285.1700; 119.0358
7	9.804	[M-H]^−^	203.0824	1.48	C_11_H_12_N_2_O_2_	204.09	L-Tryptophan	142.0661; 116.0504
8	10.566	[M-H]^−^	209.0454	−0.48	C_10_H_10_O_5_	210.18	5-Hydroxyferulic acid	165.0573; 119.0499; 93.0329
9	11.264	[M-H]^−^	593.1489	−0.17	C_27_H_30_O_15_	594.52	Nicotiflorin	593.1497; 473.1072; 383.0771; 353.0663; 325.0713
10	11.918	[M-H]^−^	563.1383	−4.08	C_26_H_28_O_14_	564.49	Schaftoside	503.1185; 383.0781; 353.0673
11	12.885	[M-H]^−^	577.1543	−3.47	C_27_H_30_O_14_	578.52	Violanthin	577.1543; 457.1124; 383.0767; 353.0666
12	13.057	[M-H]^−^	549.1597	−3.10	C_26_H_30_O_13_	550.51	Licuraside	549.1604; 429.0986; 255.0666; 135.0084
13	13.364	[M-H]^−^	549.1594	−3.64	C_26_H_30_O_13_	550.51	Liquiritin apioside	549.1582; 255.0651; 135.0084
14	13.641	[M-H]^−^	417.1175	−3.84	C_21_H_22_O_9_	418.39	Liquiritin	255.0638; 135.0075; 119.0490; 91.0184
15	16.301	[M-H]^−^	433.114	0.00	C_21_H_22_O_10_	434.39	Naringenin 7-glucoside	271.0610; 151.0026
16	17.927	[M-H]^−^	549.1593	−3.82	C_26_H_30_O_13_	550.51	Isoliquiritin apioside	549.1606; 255.0663; 135.0087
17	18.213	[M+HCOO]^−^	475.1249	0.63	C_22_H_22_O_9_	430.4	Ononin	267.0657; 252.0419
18	18.854	[M-H]^−^	417.1184	−1.68	C_21_H_22_O_9_	418.39	Isoliquiritin	417.1215; 255.0655; 148.0165; 135.0085; 119.0494
19	20.914	[M-H]^−^	255.0654	−3.53	C_15_H_12_O_4_	256.25	Liquiritigenin	135.0080; 119.0500; 91.0193
20	23.105	[M-H]^−^	823.409	−3.89	C_42_H_64_O_16_	824.95	Licorice saponin J2	823.4111; 351.0558; 193.0362
21	24.358	[M-H]^−^	373.1648	−2.41	C_21_H_26_O_6_	374.17	Hexahydrocurcumin	179.0715; 164.0463
22	25.376	[M-H]^−^	895.3939	−3.35	C_44_H_64_O_19_	896.97	Uralsaponin F	895.3935; 351.0566
23	26.957	[M-H]^−^	983.4464	−2.95	C_48_H_72_O_21_	985.07	Licorice saponin A3	983.4455; 821.3948; 351.0570
24	27.26	[M-H]^−^	1025.456	−3.41	C_50_H_74_O_22_	1027.109	22-Acetoxyl-rhaoglycyrrhizin	1025.4576; 497.1117
25	28.637	[M-H]^−^	879.3987	−3.75	C_44_H_64_O_18_	880.97	22β-Acetoxyl glycyrrhizic acid	879.3966; 351.0552
26	31.954	[M-H]^−^	819.3787	−2.68	C_42_H_60_O_16_	820.92	Licorice saponin E2	818.3758; 351.0551
27	32.062	[M-H]^−^	837.3885	−3.46	C_42_H_62_O_17_	838.93	Licorice saponin G2	837.3851; 351.0550
28	32.866	[M-H]^−^	863.4042	−3.36	C_44_H_64_O_17_	864.97	22-β-Acetoxyglycyrrhetaldehyde	863.4068; 351.0560
29	33.204	[M-H]^−^	967.4498	−4.75	C_48_H_72_O_20_	969.07	Rhaoglycyrrhizin	967.4524; 497.1136
30	34.335	[M-H]^−^	821.3933	−3.90	C_42_H_62_O_16_	822.93	Glycyrrhizin	821.3911; 351.0543; 193.0346
31	35.308	[M-H]^−^	807.4156	−1.98	C_42_H_64_O_15_	808.95	Licoricesaponin B2	807.4160; 351.0574
32	35.896	[M-H]^−^	821.3915	−6.09	C_42_H_62_O_16_	822.93	Uralsaponin B	821.3944; 351.0571; 193.0346
33	35.947	[M-H_2_O+H]^+^	277.1821	8.30	C_17_H_26_O_4_	294.18	6-Gingerol	177.0917; 145.0654; 137.0601; 117.0705
34	36.545	[M-H]^−^	821.393	−4.26	C_42_H_62_O_16_	822.93	Licoricesaponin K2	821.3953; 351.0567; 193.0366
35	36.954	[M+HCOO]^−^	823.4094	−3.40	C_41_H_62_O_14_	778.92	Apioglycyrrhizin	823.4100; 777.4044; 351.0574
36	37.944	[M+H]^+^	231.1403	9.95	C_15_H_18_O_2_	230.13	Atractylenolide Ⅰ	161.0614; 157.0998; 91.0539
37	38.246	[M-H]^−^	805.3974	−5.21	C_42_H_62_O_15_	806.93	Licoricesaponin C2	805.3996; 351.0562; 193.0358
38	38.664	[M-H]^−^	367.1178	−2.45	C_21_H_20_O_6_	368.38	Glycycoumarin	367.1164; 309.0396; 297.0401; 201.0182
39	39.273	[M-H]^−^	355.1543	−2.25	C_21_H_24_O_5_	356.41	Glyasperin C	323.1291; 203.0718; 135.0447
40	40.102	[M-H]^−^	353.105	5.38	C_20_H_18_O_6_	354.35	Licoisoflavone A	353.1025; 297.0398; 284.0313; 269.0445
41	40.391	[M-H]^−^	645.363	−2.17	C_36_H_54_O_10_	646.81	Glycyrrhetinic acid 3-O-mono-beta-D-glucuronide	645.3624; 469.3118; 113.0250
42	40.83	[M-H]^−^	381.1339	−1.31	C_22_H_22_O_6_	382.41	Glycyrin	351.0880; 323.0928; 201.0165
43	42.257	[M-H]^−^	365.1029	−0.55	C_21_H_18_O_6_	366.36	Glycyrol	365.1017; 295.0250
44	43.826	[M + H]^+^	233.1558	9.44	C_15_H_20_O_2_	232.15	AtractylenolideⅡ	233.1557; 187.1494; 159.0820; 105.0707
45	44.587	[M + H]^+^	277.1805	0.36	C_17_H_24_O_3_	276.17	6-Shogaol	137.0606; 122.0367; 94.0420
46	46.668	[M-H]^−^	483.3114	0.62	C_30_H_44_O_5_	484.32	Poricoic acid B	483.3106; 409.2734; 211.1467
47	48.244	[M-H]^−^	497.3245	−4.42	C_31_H_46_O_5_	498.33	Poricoic acid A	497.3235; 423.2791
48	49.519	[M-H]^−^	469.3368	9.59	C_30_H_46_O_4_	470.68	16α-Hydroxydehydrotrametenolic acid	469.3308; 425.3363

### 3.2 GJLZ decoction treatment improved MCD–induced hepatic steatosis

Compared to the ND group, the MCD group exhibited a significantly lower body weight, along with a markedly higher liver-to-body weight ratio (Figures 1B, C). GJLZ-S intervention markedly reduced liver-to-body weight ratio (Figure 1C). Moreover, MCD diet significantly induced severe steatosis, hepatic inflammation, and ballooning degeneration, whereas GJLZ-S intervention significantly reduced hepatic steatosis NAS, and liver TG level (Figures 1D–F). The intervention GJLZ-L and GJLZ-S both markedly reduced the level of alanine transaminase, not aspartate transaminase (Figures 1G, H). In addition, GJLZ-S intervention also markedly reduced the levels of TG and nonesterified fatty acid (NEFA) (Figures 1I, J). This indicated that GJLZ-S significantly improved MCD–induced hepatic steatosis. IHC of F4/80 showed that GJLZ-S significantly reduced macrophage infiltration, implying alleviation of hepatic inflammation (Figure 1K).

### 3.3 GJLZ decoction remodeled gut flora in mice induced by MCD

**FIGURE 3:**
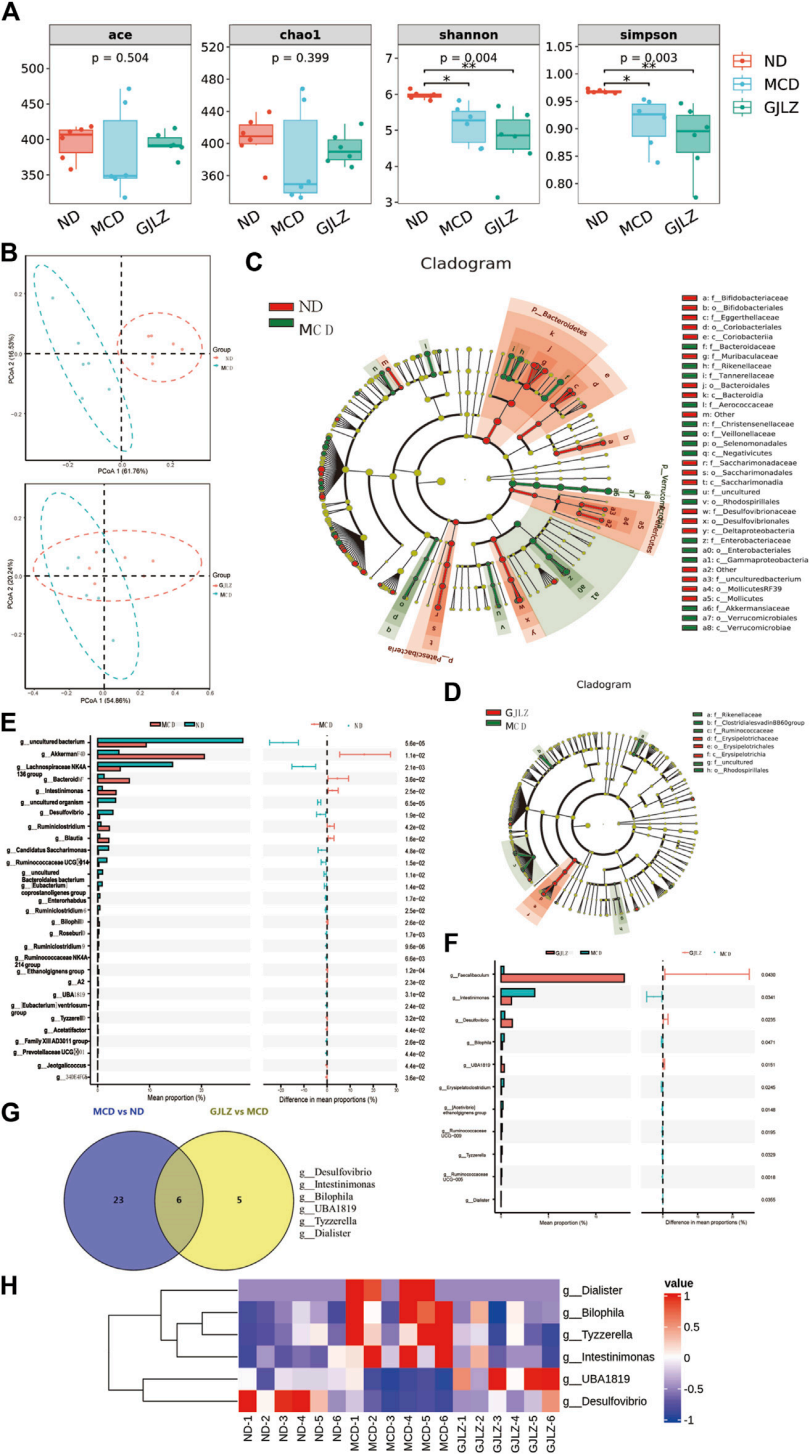
GJLZ decoction treatment remodeled gut flora in mice with MCD-induced hepatic steatosis. **(A)** Alpha diversity and **(B)** beta diversity were performed. **(C,D)** Cladograms of gut flora were analyzed in MCD group versus normal diet (ND) and GJLZ group versus MCD group. **(E,F)** Differential gut floras were shown in MCD group versus ND group and GJLZ decoction group versus MCD group. **(G,H)** Gut flora common was performed among three groups. Data are presented as the mean ± SD (n = 6). ^*^ *p* < 0.05, ^**^ *p* < 0.01.

In NASH, the gut-hepatic axis plays a significant role. Dysbiosis in gut microbiota can lead to increasement of intestinal permeability, allowing harmful substances to reach the liver. This can trigger inflammation, liver cell damage, and the progression of NASH (Boursier et al., 2016). Thus, 16S rDNA sequencing was employed to identify and examine comprehensive alterations of gut flora at family and genus level, and the abundances belonging to *Muribaculaceae*, *Erysipelotrichaceae*, *Akkermansia*, and *Faecalibaculum* differed between the three groups (Supplementary Figures S1A, B). The ecological variety of the gut flora was analyzed by utilizing alpha diversity indexes. The MCD group and GJLZ decoction group had significantly lower Simpson and Shannon indexes compared with that in the ND group, not ACE and Chao1 indexes (Figure 3A). To investigate structural variation in the gut flora, PCoA was performed to determine beta diversity (Figure 3B). The analysis of the gut flora composition was conducted (Supplementary Figure S1C). The analysis of gut flora cladograms was also performed (Figures 3C, D), and differences were identified, including in *Rikenellaceae*, *Clostridiales vadinBB6*, *Ruminococcaceae*, *Erysipelotrichaceae*, and *Rhodospirillales*. Differences in abundances of flora were analyzed with a *P*-value < 0.05. In total, flora abundance of 29 genera differed significantly between the MCD and the ND groups, and that of 11 genera differed significantly between the GJLZ and the MCD groups (Figures 3E, F; Supplementary Material S1, S2). The KEGG analysis focused on pathways, including the metabolism pathways of carbohydrate, amino acid and energy, and membrane transport (Supplementary Figures S1D, E). In total, the abundances of six genera were overlapped in all groups, namely *Desulfovibrio*, *Intestinimonas*, *Bilophila*, *UBA1819*, *Tyzzerella*, and *Dialister* (Figure 3G). The data indicate a significant increase in the abundance of *Dialister*, *Bilophila*, *Tyzzerella*, and *Intestinimonas* in the MCD group, which was notably reversed by GJLZ treatment. Conversely, the abundance of *UBA1819* and *Desulfovibrio* was significantly reduced in the MCD group, but GJLZ treatment significantly elevated their levels (Figure 3H).

### 3.4 GJLZ decoction treatment restored the alteration of metabolites

**FIGURE 4:**
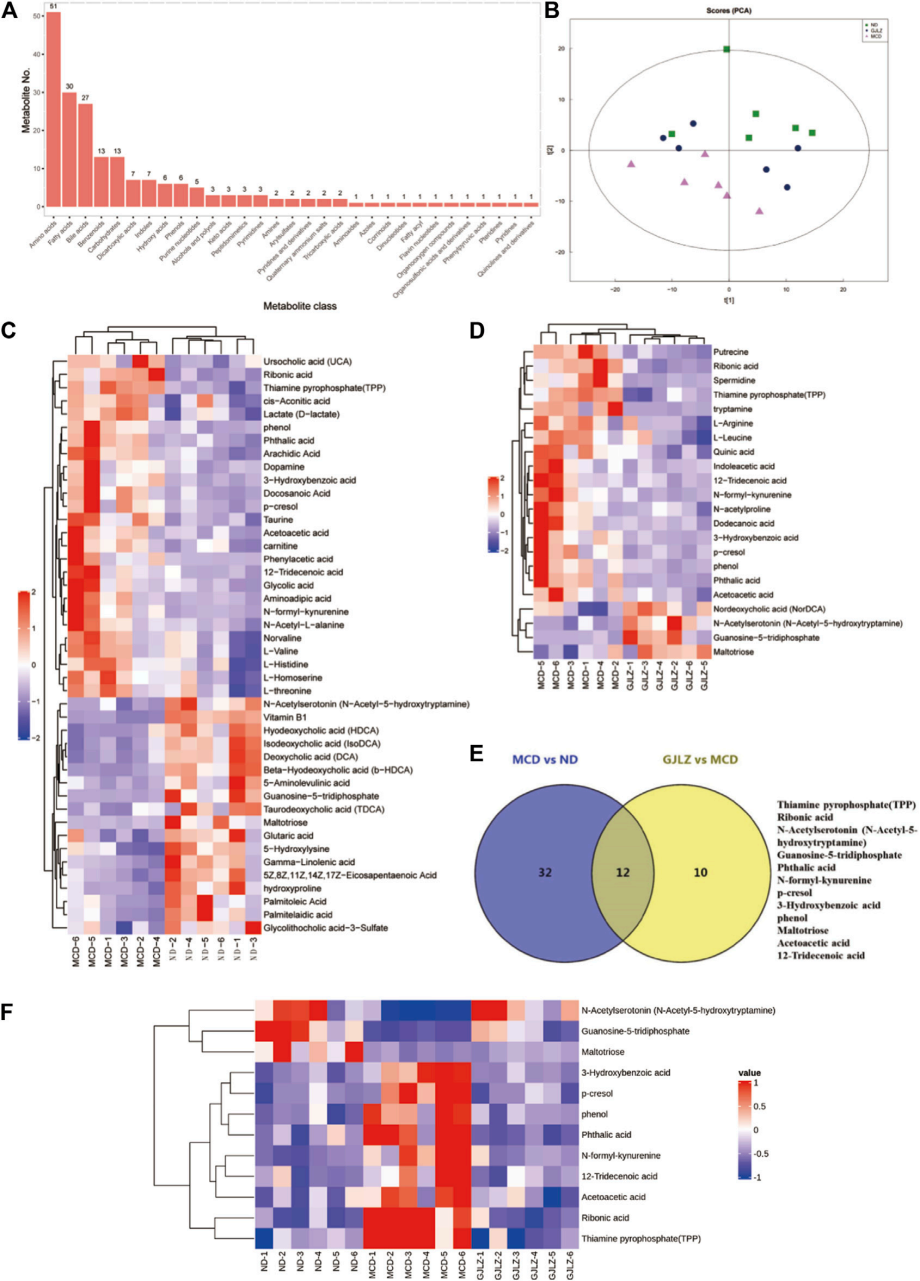
GJLZ decoction treatment restored MCD diet–induced alteration of metabolites. **(A)** Main identified metabolites were shown. **(B)** Principal component analysis of three groups was performed. **(C)** Differential metabolites in MCD group versus ND group, and **(D)** Differential metabolites in GJLZ decoction group versus MCD group were found. **(E,F)** Common metabolites among three groups were shown (n = 6).

Targeted metabolomics of feces samples was performed, primarily identifying amino acids, bile acids, and fatty acids (Figure 4A). Distinct differences of metabolites among the ND, MCD, and GJLZ decoction groups were analyzed (Figure 4B; Supplementary Figure S2A). In total, 44 significant differential metabolites were found in the MCD group versus the ND group, and 22 significant differential metabolites were discovered in the GJLZ decoction group versus the MCD group (Figures 4C, D; Supplementary Material S3). These were mainly ATP-binding cassette transporters or involved in processes such as amino acid biosynthesis and aminoacyl-tRNA biosynthesis (Supplementary Figure S2B). In total, 12 metabolites were overlapped in the all groups using the Venn diagram (Figure 4E). The concentrations of N-Acetylserotonin (N-Acetyl-5-hydroxytryptamine), guanosine-5-triphosphate, and maltotriose were markedly diminished in the MCD group, whereas GJLZ administration significantly elevated these levels. Additionally, the concentrations of 3-Hydroxybenzoic acid, p-cresol, phenol, phthalic acid, N-formyl-kynurenine, 12-Tridecenoic acid, acetoacetic acid, ribonic acid, and thiamine pyrophosphate (TPP) were significantly increased in the MCD group, with GJLZ treatment effectively reversing these elevations (Figure 4F).

**FIGURE 5:**
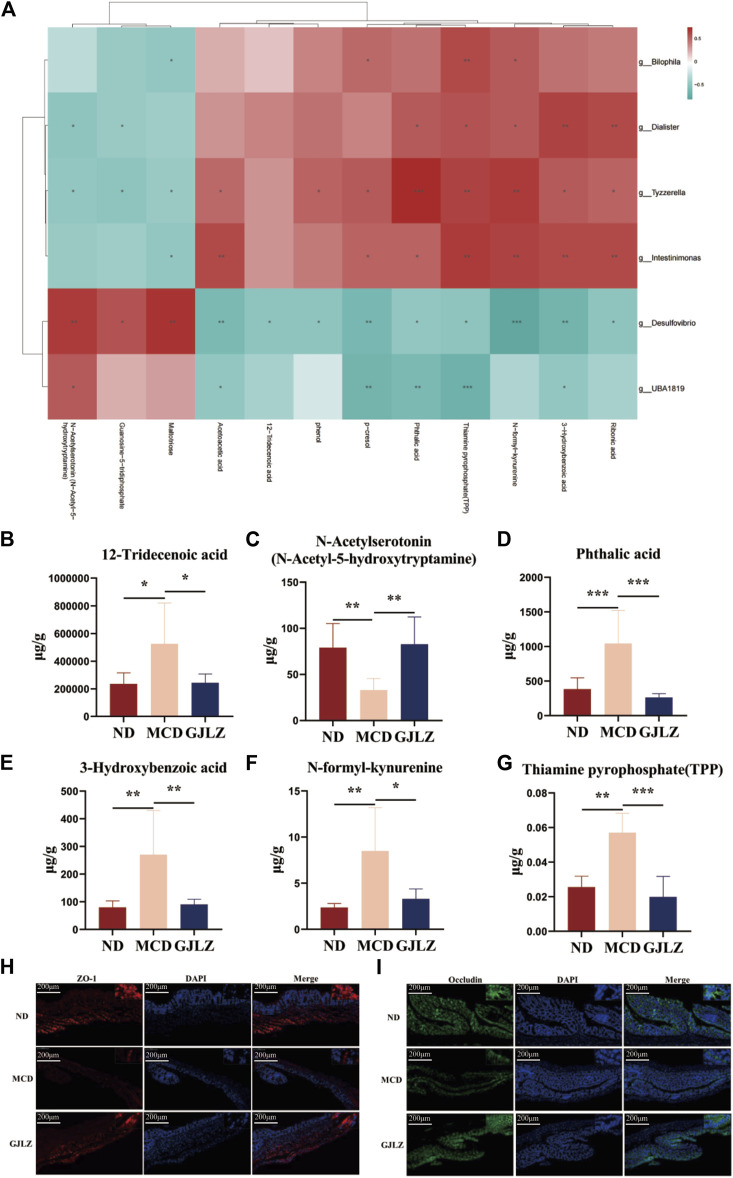
Correlational analysis of gut flora and metabolites. **(A)** Correlational analysis of gut flora and metabolites was showed among the three groups. Levels of **(B)** 12-tridecenoic acid, **(C)** N-acetylserotonin, **(D)** phthalic acid, **(E)** 3-hydroxybenzoic acid, **(F)** N-formyl-kynurenine, and **(G)** thiamine pyrophosphate were shown. **(H,I)** Levels of ZO-1 and occludin in mice were determined. Data are presented as the mean ± SD (n = 6). ^*^ *p* < 0.05, ^**^ *p* < 0.01, ^***^ *p* < 0.001.

In order to unveil the interrelation between gut flora and metabolites, correlations between metabolites and gut flora were identified (Figure 5A). The levels of 12-tridecenoic acid and five strongly correlated metabolites were analyzed. The N-acetylserotonin level was markedly lower in the MCD group, and markedly higher in the GJLZ decoction group, whereas the levels of other five metabolites were markedly higher in the MCD group than that in the GJLZ decoction group. The 12-tridecenoic acid level was highest in feces samples among the three groups (Figures 5B–G). Moreover, the intestinal permeability of the mice was also assessed. There was a significant difference between the MCD and ND groups in terms of ZO-1 and occludin levels, whereas GJLZ intervention significantly promoted their levels (Figures 5H, I). These results indicated that intestinal permeability was damaged in the MCD group, whereas GJLZ intervention could protect intestinal permeability.

### 3.5 12-Tridecenoic acid aggravated hepatic steatosis

**FIGURE 6:**
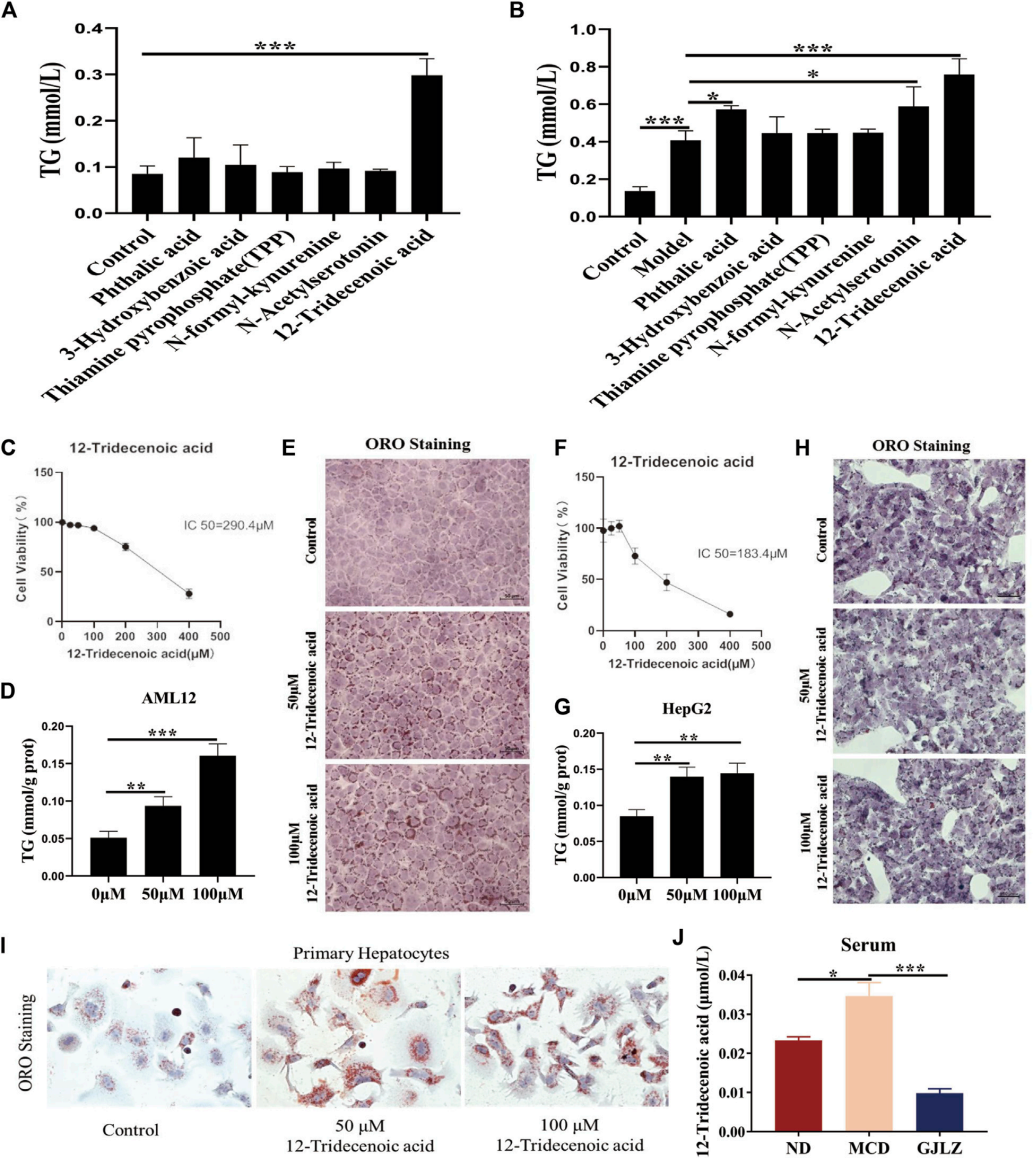
12-Tridecenoic acid aggravated hepatic steatosis. **(A,B)** TG level was measured in AML12 cells treated with metabolites with or without free fatty acids. **(C)** Effect of 12-tridecenoic acid on viability of AML12 cells was performed. **(D,E)** TG and oil red O staining in AML12 cells treated with 12-tridecenoic acid were performed. **(F)** Effect of 12-tridecenoic acid on viability of human hepatocellular carcinoma (HepG2) cells was performed. **(G,H)** TG and oil red O staining in HepG2 cells treated with 12-tridecenoic acid were performed. **(I)** Effect of 12-tridecenoic acid on primary hepatocytes was performed. **(J)** Serum level of 12-tridecenoic acid in mice was measured. Data are presented as the mean ± SD (n = 3). ^*^ *p* < 0.05, ^**^ *p* < 0.01, ^***^ *p* < 0.001.

Six metabolites on the effect of hepatic steatosis were verified. 12-Tridecenoic acid treatment (100 μM) markedly increased the TG level in AML12 with or without FFA treatment. Treatment with 100 μM phthalic acid and N-acetylserotonin markedly increased the TG level in AML12 cells treated with FFAs (Figures 6A, B), indicating that 12-tridecenoic acid may be key in hepatic steatosis. First, the cytotoxicity of 12-tridecenoic acid for AML12 cells was verified using CCK-8. It had an IC_50_ value of 290.4 μM (Figure 6C). Treatment with 50 μM or 100 μM 12-tridecenoic acid significantly promoted steatosis in AML12 cells (Figures 6D, E). The IC_50_ of 12-tridecenoic acid for HepG2 cells was 183.4 μM (Figure 6F). Treatment with 50 or 100 μM 12-tridecenoic acid significantly promoted steatosis in HepG2 cells (Figures 6G, H). In addition, primary hepatocytes were extracted and treated with 12-tridecenoic acid, and results indicated that treatment with 50 μM or 100 μM 12-tridecenoic acid significantly promoted steatosis (Figure 6I). To further explore how 12-tridecenoic acid in the gut affected steatosis of liver, level of 12-tridecenoic acid in serum were verified. The result showed that the level of 12-tridecenoic acid in serum was markedly increased in the MCD group, whereas GJLZ intervention markedly reduced its level (Figure 6J). These results demonstrated that MCD diet impaired intestinal permeability, and caused 12-tridecenoic acid in the intestine to enter the serum, inducing hepatic steatosis, whereas GJLZ could improve steatosis by regulating the gut-liver axis.

### 3.6 GJLZ decoction treatment alleviated steatosis by 12-tridecenoic acid regulating ACC activity

**FIGURE 7:**
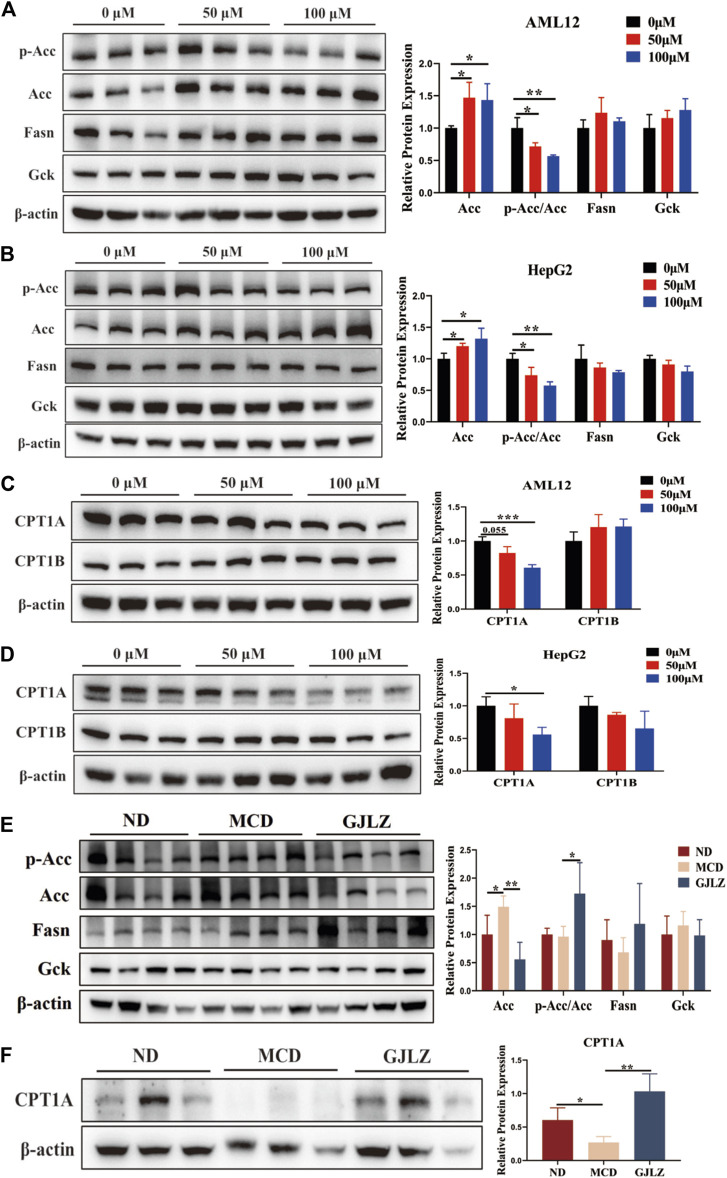
GJLZ decoction treatment alleviated steatosis by regulating acetyl-coenzyme A carboxylase alpha (ACC) activity. **(A,B)** Protein levels of ACC, FASN, and GCK in AML12 cells and HepG2 cells treated with 12-tridecenoic acid were measured (n = 3). **(C,D)** Protein levels of carnitine palmitoyltransferase 1A and carnitine palmitoyltransferase 1B in AML12 and HepG2 cells treated with 12-tridecenoic acid were measured (n = 3). **(E)** Protein levels of ACC, FASN, and GCK were measured after GJLZ treatment (n = 6). **(F)** Protein levels of carnitine palmitoyltransferase 1A after GJLZ treatment were measured (n = 6). Data are presented as the mean ± SD. ^*^ *p* < 0.05, ^**^ *p* < 0.01, ^***^ *p* < 0.001.

*De novo* lipogenesis (DNL) is the primary pathway for fat synthesis, and ACC is key to increasing DNL and decreasing fatty acid β-oxidation (Ross et al., 2020; Batchuluun et al., 2022). FASN also plays a central role in DNL (Lupu and Menendez, 2006; Wang et al., 2022). Moreover, increased levels of glucose and carbohydrates promote fatty acid synthesis by enhancing acetyl-CoA production through glycolysis. GCK could regulate glucose metabolism and has implications for liver steatosis. Therefore, levels of ACC, FASN and GCK were detected to explore the mechanism of 12-tridecenoic acid on steatosis. Results showed that 12-tridecenoic acid remarkably enhanced the ACC protein level and significantly reduced phosphorylation of ACC, but not that of FASN and GCK (Figures 7A, B). ACC can limit CPT1A and carnitine palmitoyltransferase 1B (CPT1B) levels to regulate fatty acid β-oxidation. Therefore, levels of CPT1A and CPT1B in AML12 and HepG2 cells treated with 12-tridecenoic acid were analyzed. 12-tridecenoic acid markedly inhibited protein synthesis of CPT1A but not that of CPT1B (Figures 7C, D). In addition, GJLZ treatment significantly downregulated level of ACC, and markedly promoted ACC phosphorylation (Figure 7E). GJLZ treatment also significantly promoted the level of CPT1A (Figure 7F). This indicated that 12-tridecenoic acid induced hepatic steatosis by regulating the ACC–CPT1A axis, whereas GJLZ could improve steatosis by 12-tridecenoic acid regulating ACC activity.

### 3.7 Effect of 12-tridecenoic acid on steatosis was reversed by ACC inhibition

To explore the mechanism of 12-tridecenoic acid on steatosis, ACC inhibition was performed using an ACC inhibitor. Results showed that treatment with 50 μM or 100 μM 12-tridecenoic acid significantly increased the TG levels in AML12 and HepG2 cells, and this effect was reversed by ACC inhibition (Supplementary Figure S3). These results indicated that 12-tridecenoic acid induced steatosis by regulating ACC activity.

## 4 Discussion

Our study in mice with MCD–induced NASH showed that GJLZ decoction treatment alleviated the hepatic steatosis by regulating the 12-tridecenoic acid–mediated ACC–CPT1A axis to decrease hepatic DNL. Because NAFLD is a multisystem disease, its pathogenesis remains unclear. However, FFAs are central to the onset of NAFL and NASH (Mota et al., 2016). Even though the absolute level of DNL in humans is lower than that in rodents, the hepatic DNL level has an important influence on human NAFLD (Deprince et al., 2020). Excessive DNL leads to an increased production of fatty acids within the liver, which can contribute to this fat accumulation and worsen the progression of NAFLD. Furthermore, elevated DNL can also lead to the production of pro-inflammatory lipids (Heida et al., 2021; Gluais-Dagorn et al., 2022). As the first step rate-determining enzyme in DNL, which promotes the adenosine triphosphatase–dependent carboxylation (Musso et al., 2009), ACC is key to increasing DNL and decreasing fatty acid β-oxidation (Du et al., 2022). A study identified that an ACC1/2 inhibitor called TOFA could completely inhibit hepatic DNL (Harada et al., 2007). A study involving 126 individuals diagnosed with NASH found that the daily intake of 20 mg firsocostat, an inhibitor of ACC, resulted in a significant decrease of 29% in the amount of fat in the liver (Alkhouri et al., 2020). Moreover, PF-05221304, a novel ACC1/2 inhibitor, could significantly reduce liver fat with monotherapy doses ≥10 mg (Calle et al., 2021). In this study, the 12-tridecenoic acid level was significantly higher in mice with MCD–induced hepatic steatosis. Moreover, 12-tridecenoic acid contributed to hepatic steatosis in AML12 and HepG2 cells, and significantly promoted ACC activity. A further study found that the effect of 12-tridecenoic acid on steatosis was reversed by ACC inhibition with TOFA, indicating that 12-tridecenoic acid promoted hepatic DNL by increasing ACC activity. In the study, we also showed that GJLZ treatment significantly reduced the level of 12-tridecenoic acid, and significantly inhibited ACC activity. In addition, CPT1A on the mitochondrial membrane is crucial to the free transportation of medium- and long-chain fatty acids into mitochondria. The effectiveness of fatty acid oxidation is directly linked to the activity of CPT1A (Ribas and Vargas, 2022). Studies have demonstrated that ACC could inhibit CPT1A activity to regulate fatty acid oxidation (Fang et al., 2019; Yao et al., 2020). In our study, these results showed that 12-tridecenoic acid significantly inhibited CPT1A expression, whereas GJLZ decoction significantly promoted CPT1A expression. Therefore, our results demonstrated that GJLZ decoction could hepatic steatosis by regulating 12-tridecenoic acid–mediated ACC–CPT1A axis. However, our findings also indicated that there was no significant difference in TG and NEFA levels between the ND group and the MCD group, with a slight reduction observed. We hypothesize that this phenomenon may be attributed to the MCD diet’s inhibition of lipid export, which occurs due to the depletion of phosphatidylcholine, an essential component of very low-density lipoprotein. This deficiency leads to increased hepatic lipid accumulation and a corresponding decrease in serum lipid levels. As a result, TG and NEFA concentrations were found to be reduced in the MCD group.

Gut flora plays significant roles in maintaining host health by interacting with the immune system, influencing metabolism, and participating in various physiological processes, including NAFLD (Aron-Wisnewsky et al., 2020; Behary et al., 2021; Wang et al., 2021). Therefore, intestinal flora dysbiosis is crucial in NAFLD. Studies have shown that individuals with NAFLD exhibit alterations of gut flora compared with healthy individuals, including a decrease in beneficial bacteria (such as *Bacteroidetes*, *Faecalibacterium prausnitzii*, *Desulfovibrio*, *Lactobacillus lactis*, *bifidobacterium*) and an increase in potentially harmful bacteria (*Proteobacteria*, *Escherichia*) (Hong et al., 2021; Long et al., 2021; Yu et al., 2021; Chen et al., 2022; Hu et al., 2022; Hui et al., 2022; Xin et al., 2022; Shin et al., 2023). *Desulfovibrio* attenuates NAFLD in mice by suppressing *FASN* and *CD36* expression (Hong et al., 2021). *Desulfovibrio* promotes the secretion of hydrogen sulfide and enhances insulin sensitivity in mice with NAFLD via the AKT pathway (Chen et al., 2022). *UBA1819*, from the *Ruminococcaceae* family, attenuates hepatic steatosis (Milton-Laskibar et al., 2022). In our study, the levels of *Desulfovibrio* and *UBA1819* were markedly lower in the MCD group, and GJLZ treatment significantly promoted their levels, which was consistent with the previous studies (Hong et al., 2021; Chen et al., 2022; Milton-Laskibar et al., 2022). This indicates that *Desulfovibrio* and *UBA1819* may be essential in NAFLD progression. Furthermore, the *Desulfovibrio* level was significantly negatively correlated with the 12-tridecenoic acid level, which promoted hepatic steatosis by regulating the ACC–CPT1A axis. A previous study has shown that *Desulfovibrio* can attenuate NAFLD by suppressing *FASN* expression, whereas our results showed that *Desulfovibrio* may attenuate NAFLD by mediating the 12-tridecenoic acid-ACC-CPT1A axis. Organic acids present in traditional Chinese medicine can create an acidic environment in the intestinal tract, thereby reducing the intestinal pH value and fostering conditions conducive to the growth and colonization of intestinal probiotics. GJLZ, in particular, contains gluconic acid, citric acid, poria acid, and other organic acids, which may play a role in regulating the proliferation of intestinal probiotics. Furthermore, the study demonstrated that pachycoia oligosaccharides could decrease the *Firmicutes* to *Bacteroidetes* ratio by modulating the intestinal microbiota, thereby ameliorating disorders of glucose and lipid metabolism (Zhu et al., 2022). In our study, the genera *Dialister*, *Bilophila*, and *Intestinimonas*, all members of the *Firmicutes* phylum, exhibited elevated expression levels in mice subjected to an MCD diet. However, administration of GJLZ markedly attenuated their expression. Moreover, glycyrrhiza polysaccharides have been shown to significantly upregulate *Ruminococcus*, thereby enhancing intestinal health (Qiao et al., 2022). Specifically, within our study, the abundance of *UBA1819*, a member of the *Ruminococcaceae* family, was significantly diminished in the MCD group, whereas GJLZ administration substantially increased UBA1819 levels. This modulation of the intestinal microbiota subsequently influences the production of medium- and long-chain fatty acids, which further contributes to its functional benefits. Based on these findings, we hypothesize that the active components in GJLZ may modulate alterations in the intestinal microbiota, leading to changes in metabolite profiles and ultimately ameliorating liver steatosis. However, this study has several limitations. First, while current research has yet to directly demonstrate that GJLZ modulates intestinal flora and differential metabolites to ameliorate liver degeneration, future studies will aim to further investigate the effects of GJLZ and its active constituents on intestinal flora and metabolites. This will be undertaken with the objective of elucidating the specific mechanisms underlying the therapeutic actions of GJLZ. Second, how may *Desulfovibrio* regulate the 12-tridecenoic acid level is unclear. Third, gut flora and metabolites from feces were tested in this study. It is unclear whether gut flora and metabolites in the intestinal tract influence NASH *in vivo*. Finally, 12-tridecenoic acid is a monounsaturated fatty acid with a chain length of 13 carbon atoms. Its biological functions and effects are not extensively studied compared to other more abundant fatty acids. Although our results demonstrated that 12-tridecenoic acid contributed to hepatic steatosis, additional experiments are required to further validate its functionality.

## 5 Conclusion

**FIGURE 8:**
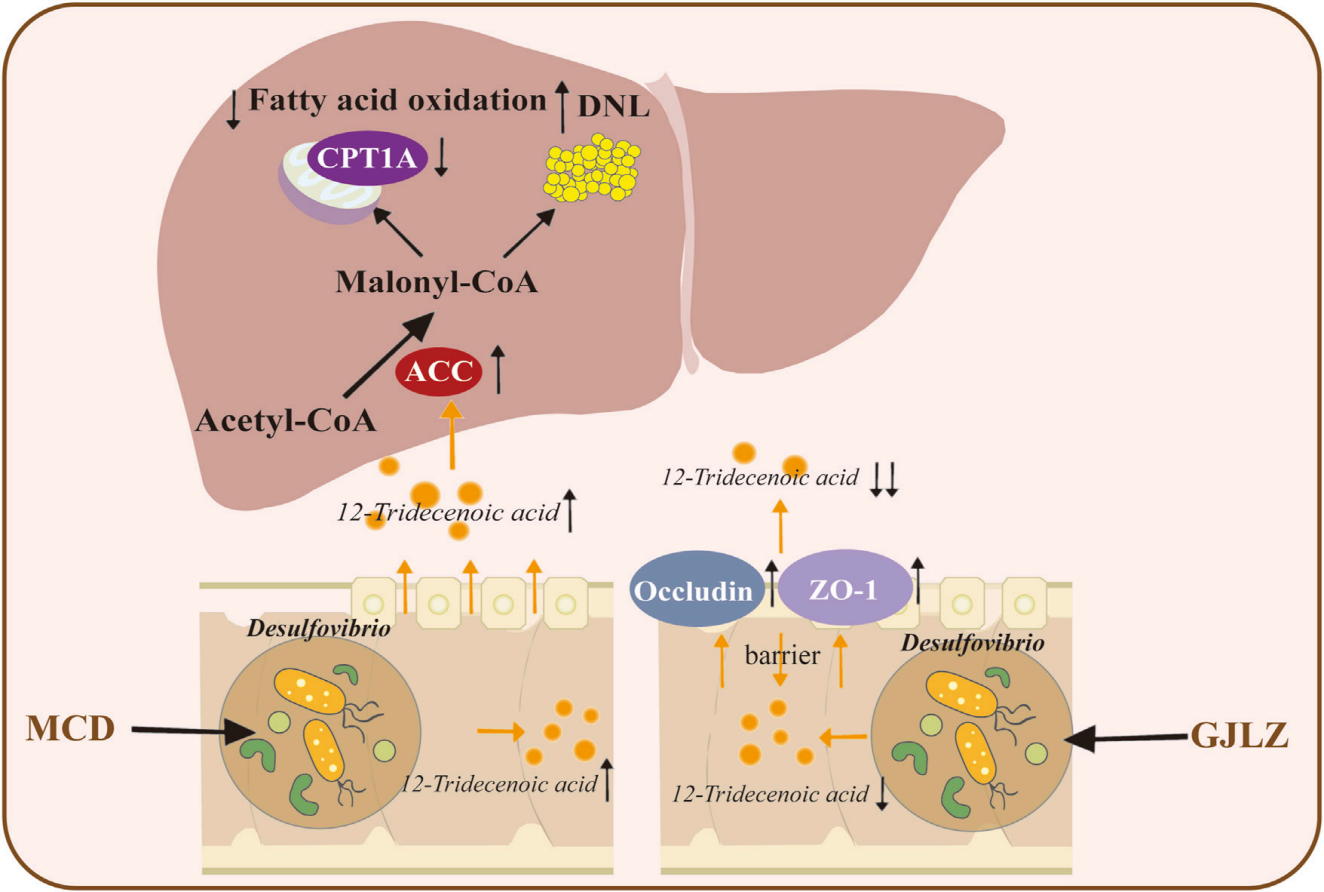
Graphical summary of the study. GJLZ decoction alleviates non-alcoholic steatohepatitis by modulating gut flora-mediated 12-tridecenoic acid inhibition.

12-tridecenoic acid aggravated hepatic steatosis by regulating the ACC–CPT1A axis, and GJLZ decoction effectively reduced the 12-tridecenoic acid level and improved hepatic steatosis. This demonstrated that treatment with GJLZ decoction improved NASH through gut flora–mediated 12-tridecenoic acid inhibition (Figure 8).

## Data Availability

Raw sequencing data of the present study are available at https://www.ncbi.nlm.nih.gov/sra/PRJNA1148162 (BioProject ID: PRJNA1148162).

## Supplementary material

SUPPLEMENTARY FIGURE S1 Analysis of gut flora among three groups.

SUPPLEMENTARY FIGURE S2 Analysis of metabolites among three groups.

SUPPLEMENTARY FIGURE S3 Effect of 12-tridecenoic acid on steatosis reversed by ACC inhibition.

SUPPLEMENTARY FIGURE S4 Effects of N-acetylserotonin on LPS-induced MODE-K cells.

SUPPLEMENTARY MATERIAL S1 The abundance of 29 significantly differed flora between the MCD and the ND groups.

SUPPLEMENTARY MATERIAL S2 The abundance of 11 significantly differed flora between the GJLZ and the MCD groups.

SUPPLEMENTARY MATERIAL S3 Differential metabolites discovered in the MCD group versus the ND group, and the GJLZ group versus the MCD group.

## Abbreviations

ACC, acetyl-coenzyme A carboxylase alpha; AML12, alpha mouse liver 12; CPT1A, carnitine palmitoyltransferase 1A; CPT1B, carnitine palmitoyltransferase 1B; DNL, *de novo* lipogenesis; FFA, free fatty acid; FASN, fatty acid synthase; GCK, glucokinase; GJLZ, *gan–jiang–ling–zhu* decoction; GJLZ-L, low-dose GJLZ decoction; GJLZ-S, standard-dose GJLZ decoction; HepG2, human hepatocellular carcinoma; MCD, methionine- and choline-deficient; NAS, NAFLD activity score; NAFLD, nonalcoholic fatty liver disease; ND, normal diet; NASH, nonalcoholic steatohepatitis; TC, total cholesterol; TCM, traditional Chinese medicine; TG, triglyceride; NEFA, nonesterified fatty acid.
