# Supplementary figures and images for: Gan-jiang-ling-zhu decoction improves steatohepatitis by regulating gut microbiota-mediated 12-tridecenoic acid inhibition
Source: Front Pharmacol. 2024 Aug 23;15:1444561. doi: 10.3389/fphar.2024.1444561 (PMC11377346; doi:10.3389/fphar.2024.1444561)

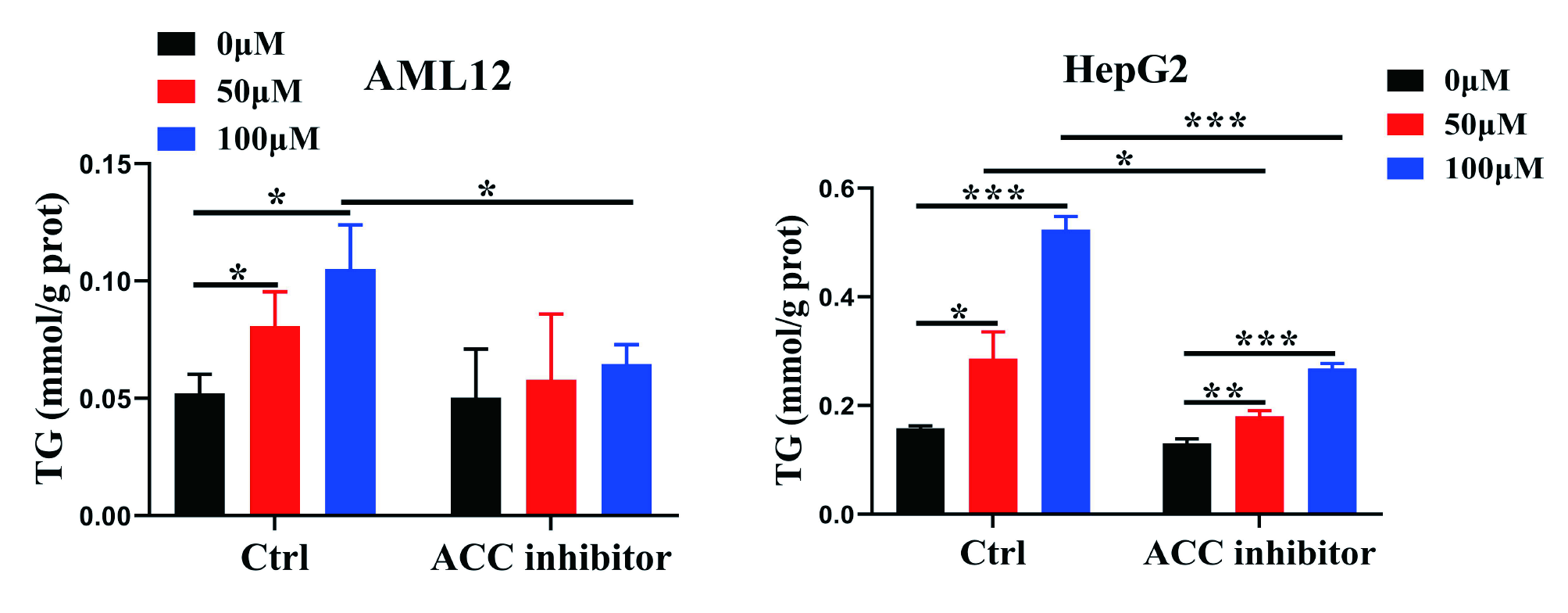

Supplement: Supplementary file 1 [file Image3.TIF]

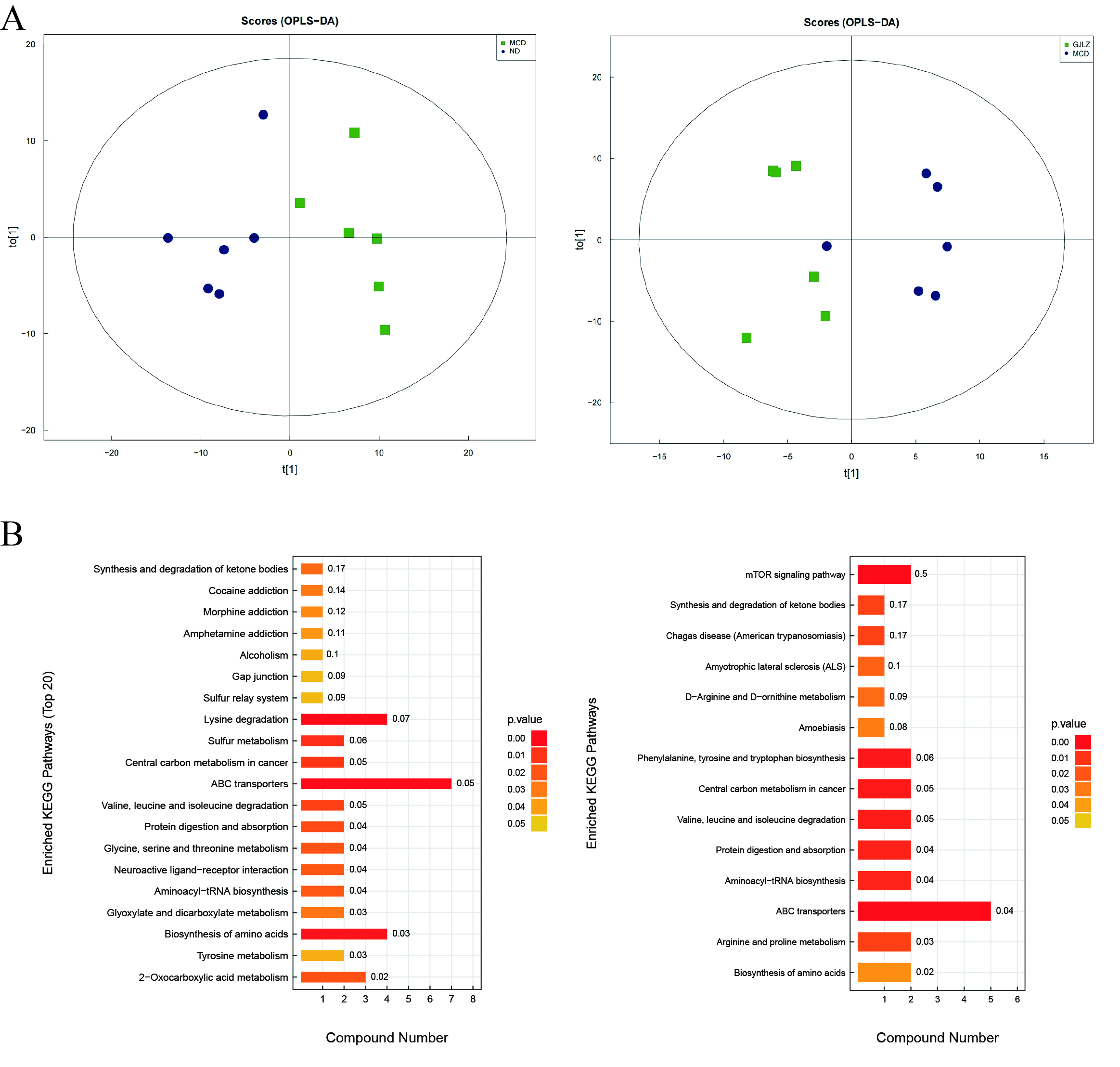

Supplement: Supplementary file 2 [file Image2.TIF]

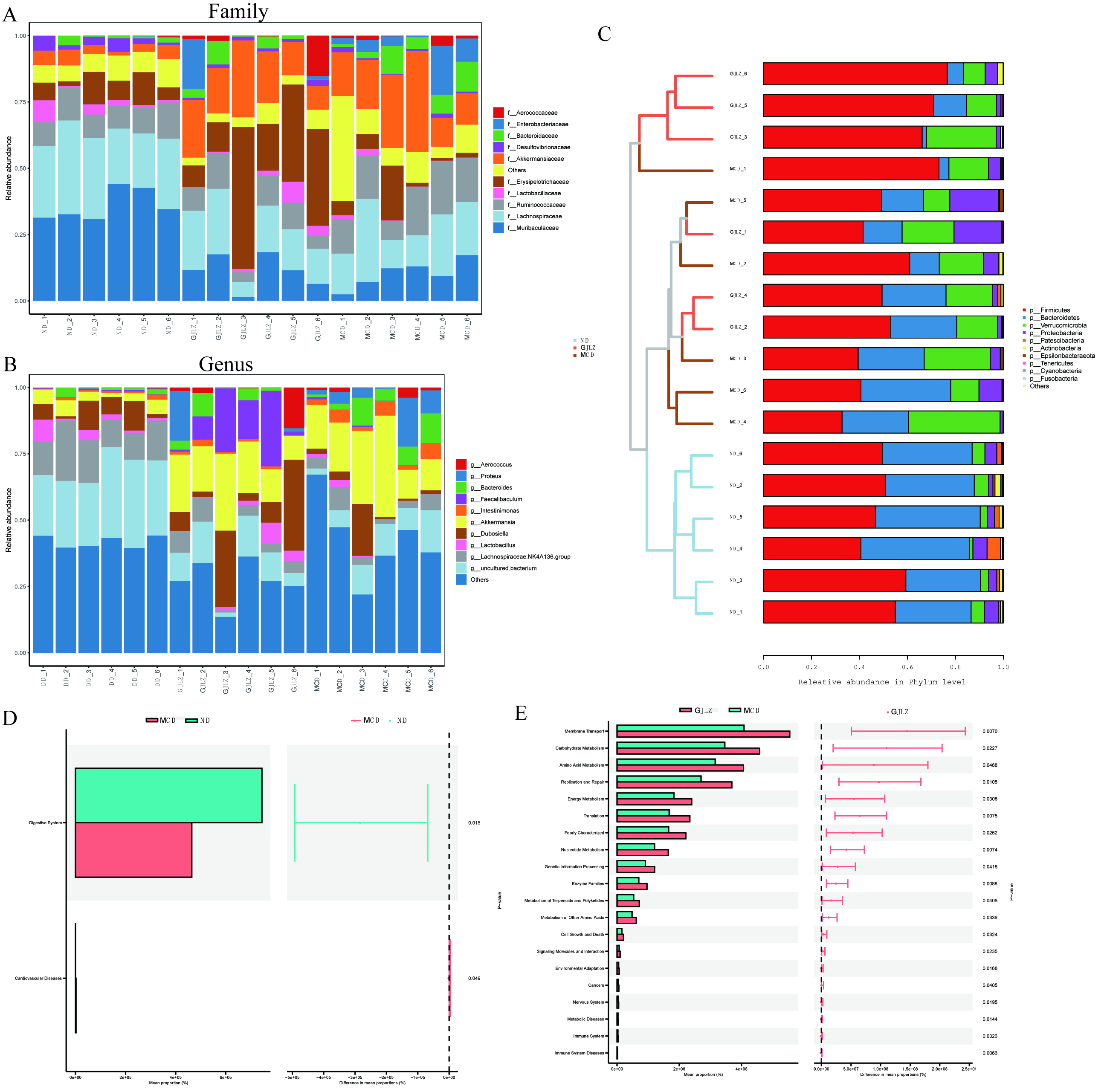

Supplement: Supplementary file 3 [file Image1.TIF]
